# List of Diseases from the 20th of February to the 20th of March; Being the Result of the Public and Private Practice of a Physician at the West End of the Town

**Published:** 1799-04

**Authors:** 


*Lift of Difeafes from the 20th of February to the 20th of March; being the refult of the public and private Practice of a Phyfician at the Weft end of the town.*

IT is worthy of remark, that notwithftanding the predominance of inflammatory complaints during the laft fix weeks, putrid fevers, and the fcarlatina anginofa, in its malignant form, have been very prevalent. The latter, more efpecially, has proved in many inftances fatal; and in thofe who recovered, it produced, after the ceffation of the fever, anafarca, fwelling of the abdomen, fwelling of the lips and parotid glands, ftrumous ophthalmia, with an eruption of the favus, and hectical fymptoms of long duration. The difeafe fpread from London to the adjacent villages, and was almoft univerfal in Sommers-town, during the month of February. R. W.

| ACUTE DISEASES. | |
|---|---|
| Catarrh — — | 37 |
| Acute Rheumatifm — | 7 |
| Inflammatory Sore Throat | 4 |
| Ophthalmia — — | 3 |
| Peripneumony — — | 2 |
| Malignant Fever — | 7 |
| Scarlatina Anginofa — | 10 |
| Meafles — — | 7 |
| Hooping Cough — | 3 |
| Small-pox — — | 3 |
| Herpes Zofter — — | 1 |
| Childbed and milk fevers | 4 |
| Acute difeafes of infants | 11 |
| Tertian — — | 2 |
| CHRONIC DISEASES. | |
| Cough and Dyfpnœa | 87 |
| Hemoptoe — — | 5 |
| Pulmonary Confumption | 12 |
| Chronic Rheumatifm — | 18 |
| Sciatica — — | 2 |
| Afthenia — — | 19 |
| Dropfy — — | 6 |
| Paralyfis — — | 6 |
| Apoplexy — — | 2 |
| Cephalæa and Hemicranium | 10 |
| Vertigo and Syncope — | 6 |
| Hyfteria — — | 3 |
| St. Vitus's Dance — | 3 |
| Dyfpepfia — — | 12 |
| Hæmatemefis — | 1 |
| Gaftrodynia — — | 7 |
| Enterodynia — — | 5 |
| Diarrhœa — — | 8 |
| Conftipatio — — | 2 |
| Scirrhus of the Liver — | 1 |

Jaundice

| | | | |
|---|---|---|---|
| Jaundice | 1 | Lichen Pilaris | 2 |
| Diabetes | 1 | Prurigo | 3 |
| Gravel and Dyfury | 3 | Lepra | 1 |
| Menorrhagia | 5 | Purpura | 1 |
| Chlorofis and Amenorrhea | 6 | Gutta Rofacea | 2 |
| Fluor Albus | 3 | Impetigo | 5 |
| Hœmorrhoids | 2 | Ecthyma | 1 |
| Tabes Mefenterica | 3 | Itch | 6 |
| Scrophula | 5 | Porrigo | 8 |

